# Supplementary material for: Investigating Snake-Venom-Induced Dermonecrosis and Inflammation Using an Ex Vivo Human Skin Model
Source: Toxins (Basel). 2024 Jun 17;16(6):276. doi: 10.3390/toxins16060276 (PMC11209077; doi:10.3390/toxins16060276)
Supplement: Supplementary file 1 [file toxins-16-00276-s001.zip › toxins-2967190-supplementary.pdf]

toxins

MDPI

Supplementary Materials: Investigating Snake-Venom-Induced Dermonecrosis and Inflammation Using an Ex Vivo Human Skin Model

Table S1. Details of histological analysis of HypoSkin® model post subcutaneous venom injections.

Treatments	Timepoints	Finding description
**Untreated**	0h	Epidermis: No abnormalities were detected.<br>Dermis: Dermis at dermo-epidermal junction and the mid dermis shows small numbers of perivascular lymphocytes and plasma cells, fewer neutrophils.<br>Adnexae: Very few glands present.
E. ocellatus	6h	Epidermis: No abnormalities were detected.<br>Dermis: No abnormalities were detected.<br>Adnexae: multifocal apocrine glandular epithelium is vacuolated
	12h	Epidermis: No abnormalities were detected. Dermis: Focal disruption of the collagen in the superficial to mid dermis with hypereosinophilic collagen.<br>Adnexae: mild vacuolation of apocrine epithelium with multifocal pyknosis.
	24h	Epidermis: Focal epidermal full thickness hypereosinophilia and pyknosis, with loss of nuclei.<br>Dermis: Focal cleft at dermoepidermal junction.<br>Adnexae: No abnormalities were detected.
	36h	Epidermis: No abnormalities were detected.<br>Dermis: Focal loss of collagen in superficial dermis.<br>Adnexae: mild multifocal glands exhibit degeneration
	48h	Epidermis: No abnormalities were detected.<br>Dermis: No abnormalities were detected.<br>Adnexae: No abnormalities were detected.
B. arietans	6h	Epidermis: No abnormalities were detected.<br>Dermis: Two focal clefts in the superficial dermis; artefact.<br>Adnexae: No abnormalities were detected.
	12h	Epidermis: No abnormalities were detected.<br>Dermis: Mild focal clefts in superficial dermal collagen (artefact)<br>Adnexae: No abnormalities were detected.
	24h	Epidermis: No abnormalities were detected.<br>Dermis: Multifocal clefts with multifocal aggregates of hypereosinophilic amorphous debris (artefact).<br>Adnexae: multifocal degeneration/necrosis
	36h	Epidermis: Multifocal epidermal loss with degeneration/necrosis of cells<br>Dermis: No abnormalities were detected.<br>Adnexae: No abnormalities were detected.
	48h	Epidermis: Multifocal moderate ulceration; adjacent epidermis exhibits vacuolar degermation, pyknotic nuclei and hyereosinophilia.<br>Dermis: multifocal small clefts in the superficial dermis and the dermo-epidermal junction.<br>Adnexae: No abnormalities were detected.
N. nigricollis	6h	Epidermis: At periphery of section there is focal pyknosis<br>Dermis: No abnormalities were detected.<br>Adnexae: Focal vacuolar degeneration/pyknosis of the glandular epithelium
	12h	Epidermis: Very mild focal cleft in the superficial epidermis.<br>Dermis: No abnormalities were detected.<br>Adnexae: Mild focal glandular degeneration
	24h	Epidermis: No abnormalities were detected.<br>Dermis: Mild multifocal clefts<br>Adnexae: No abnormalities were detected.
	36h	Epidermis: No abnormalities were detected.<br>Dermis: multifocal very small clefts (artefact)

		Adnexae: Moderate multifocal degeneration of the glands
	48h	Epidermis: No abnormalities were detected.<br>Dermis: multifocal mild to moderate cleft in the superficial to mid dermis<br>Adnexae: Moderate multifocal degeneration of apocrine glands
N. haje	6h	Epidermis: No epidermis present on three slides examined (artefact)<br>Dermis: No abnormalities were detected.<br>Adnexae: Mild multifocal degeneration of glandular epithelium
	12h	Epidermis: No abnormalities were detected.<br>Dermis: Mild multifocal clefts in the superficial dermis (artefact).<br>Adnexae: Mild to moderate multifocal degeneration of glandular epithelium
	24h	Epidermis: No epidermis present on three slides examined (artefact)<br>Dermis: No abnormalities were detected.<br>Adnexae: Moderate multifocal glandular degeneration
	36h	Epidermis: No abnormalities were detected.<br>Dermis: Very mild focal cleft<br>Adnexae: mild degeneration of glandular epithelium
	48h	Epidermis: Mild multifocal erosion and ulceration. Epithelial cells at the margin of the ulceration exhibit hyereosinophilia and pyknosis<br>Dermis: No abnormalities were detected.<br>Adnexae: No abnormalities were detected.
***E.ocellatus* + SAIMR Echis monovalent**	48h	Epidermis: Focally extensive loss of epidermis with ballooning degeneration and apoptotic cells at edge of ulceration<br>Dermis: No abnormalities were detected.<br>Adnexae: Moderate multifocal degeneration in glandular epithelium
***N.nigricollis* + SAIMR polyvalent**	48h	Epidermis: No abnormalities were detected<br>Dermis: No abnormalities were detected.<br>Adnexae: Mild multifocal glandular degeneration
**Antivenom only**	48h	Epidermis: No abnormalities were detected.<br>Dermis: No abnormalities were detected.<br>Adnexae: Moderate multifocal degeneration of glandular epithelium
**Equine IgG**	48h	Epidermis: No abnormalities were detected.<br>Dermis: Mild multifocal clefts<br>Adnexae: No abnormalities were detected.

Table S2. Summary of the Morphological changes and significant results of the dynamic changes in the expression, of qualified pro-inflammatory chemokines, cytokines and growth factors of human skin biopsies injected with viper (*Echis ocellatus* and *Bitis arietans*) and elapid (*Naja nigricollis* and *N. haje*) venoms over 48 h and 120 h following the injection venom only and 48 h following antivenom intervention.

Morphological Changes	No	Mild	Moderate	Severe	
Significant values of pro-inflammatory markers	ns	*	**	***	****

		Viper venoms				Elapid venoms				Venom + antivenom experiment	
		Echis ocellatus (48 h)	*Echis ocellatus (168 h)*	*Bitis aritans (48 h)*	*Bitis aritans (168 h)*	*Naja nigricollis (48 h)*	*Naja nigricollis (168 h)*	*N.haje (48 h)*	*N.haje (168 h)*	Eocc+ AV (48 h)	N. nig+ AV (48 h)
**Morphological changes**											
**Epidermis**		No	Severe	Moderate	Severe	No	Moderate	Mild	Mild	Severe	No
**Dermis**		No	Moderate	Mild	Moderate	Moderate	Severe	No	No	No	No
**Adnexae**		No	Moderate	No	Moderate	Moderate	Severe	No	No	Mild	Mild
**pro-inflammatory chemokines, cytokines and growth factors**											
		Echis ocellatus (48 h)	*Echis ocellatus (120 h)*	*Bitis aritans (48 h)*	*Bitis aritans (120 h)*	*Naja nigricollis (48 h)*	*Naja nigricollis (120 h)*	*N.haje (48 h)*	*N.haje (120 h)*	Eocc+ AV (48 h)	N. nig+ AV (48 h)
**MCP-1**	P-value	0.5707	0.0233	0.1632	>0.9999	<0.0001	0.0139	0.0002	0.6147	0.786	<0.0001
		ns	*	ns	ns	****	*	***	ns	ns	****
	95.00 % CI	-812.7 to 178.0	-1044 to -53.25	-909.6 to 81.15	-626.8 to 363.9	-2685 to -1695	-1081 to -90.10	-1446 to -455.5	-805.3 to 185.4	-214.9 to 775.9	1208 to 2199
**RANTES**	P-value	0.8428	<0.0001	0.9512	0.0008	<0.0001	>0.9999	0.887	0.0001	>0.9999	0.9949
		ns	****	ns	***	****	ns	ns	***	ns	ns

	95.00 % CI	-1.683 to 0.4973	-16.47 to -14.29	-1.616 to 0.5636	-2.875 to -0.6950	-3.694 to -1.514	-1.144 to 1.035	-1.660 to 0.5195	-3.254 to -1.074	13.86 to 16.04	-1.540 to 0.6401
**MIP1-ALPHA**	P-value	>0.9999	<0.0001	0.8825	0.9189	<0.0001	>0.9999	0.1203	0.0139	>0.9999	<0.0001
		ns	****	ns	ns	****	ns	ns	*	ns	****
	95.00 % CI	-27.86 to 26.02	-120.7 to -66.82	-41.10 to 12.78	-40.57 to 13.32	-203.7 to -149.8	-33.28 to 20.61	-50.62 to 3.269	-58.78 to -4.891	-26.61 to 27.28	137.3 to 191.2
**MIG**	P-value	0.9352	>0.9999	<0.0001	>0.9999	0.0661	>0.9999	0.064	>0.9999	>0.9999	0.228
		ns	ns	****	ns	ns	ns	ns	ns	ns	ns
	95.00 % CI	-1.199 to 0.4047	-0.8217 to 0.7817	-2.855 to -1.252	-0.8217 to 0.7817	-1.572 to 0.03098	-0.8217 to 0.7817	-1.576 to 0.02748	-0.8217 to 0.7817	-0.5822 to 1.021	-0.1695 to 1.434
**IL1-B**	P-value	>0.9999	<0.0001	<0.0001	>0.9999	<0.0001	>0.9999	<0.0001	>0.9999	0.0327	>0.9999
		ns	****	****	ns	****	ns	****	ns	****	ns
	95.00 % CI	-0.4311 to 0.7519	-2.378 to -1.195	-18.61 to -17.42	-0.4846 to 0.6985	-1.978 to -0.7944	-0.4846 to 0.6985	-6.097 to -4.914	-0.5383 to 0.6448	0.03514 to 1.218	-0.5381 to 0.6450
**IL1-RA**	P-value	>0.9999	0.0049	0.1299	<0.0001	0.0012	<0.0001	<0.0001	<0.0001	0.9998	>0.9999
		ns	**	ns	****	**	****	****	****	ns	ns
	95.00 % CI	-86.24 to 86.17	-201.6 to -29.19	-161.0 to 11.38	-479.6 to -307.2	-221.6 to -49.21	-551.7 to -379.3	-328.7 to -156.2	-286.9 to -114.5	-116.5 to 55.89	-95.26 to 77.14
**G-CSF/CSF-3**	P-value	>0.9999	<0.0001	0.0561	<0.0001	<0.0001	0.0002	<0.0001	>0.9999	0.9999	0.013
		ns	****	ns	****	****	***	****	ns	ns	*
	95.00 % CI	-234.6 to 154.2	-1637 to -1249	-385.7 to 3.085	-862.3 to -473.4	-1458 to -1069	-567.6 to -178.8	-998.6 to -609.7	-239.1 to 149.7	-261.9 to 126.9	-426.0 to -37.22
**GM-CSF**	P-value	>0.9999	<0.0001	>0.9999	<0.0001	<0.0001	0.2659	<0.0001	0.0011	>0.9999	<0.0001
		ns	****	ns	****	****	ns	****	**	ns	****
	95.00 % CI	-5.674 to 5.466	-27.76 to -16.62	-5.913 to 5.227	-18.89 to -7.750	-60.18 to -49.04	-9.837 to 1.303	-41.98 to -30.84	-14.42 to -3.285	-5.675 to 5.465	36.87 to 48.01
**HGF**	P-value	0.0291	0.1065	<0.0001	0.8602	<0.0001	0.6141	0.0291	0.8602	0.8521	0.0079
		*	ns	****	ns	****	ns	*	ns	ns	**
	95.00 % CI	-6.020 to -0.2198	-5.497 to 0.3031	-18.21 to -12.41	-4.455 to 1.345	-9.171 to -3.371	-4.715 to 1.085	-6.020 to -0.2198	-4.455 to 1.345	-1.335 to 4.465	0.7738 to 6.574

FGF-BASIC	P-value	<0.0001	>0.9999	<0.0001	0.9175	<0.0001	0.3052	>0.9999	0.022	>0.9999	0.3052
		****	ns	****	ns	****	ns	ns	*	***	ns
	95.00 % CI	-2.082 to -0.7448	-0.8473 to 0.4902	-2.431 to - 1.093	-1.008 to 0.3300	-3.947 to -2.609	-1.167 to 0.1702	-0.7045 to 0.6330	-1.415 to -0.07749	-0.7934 to 0.5441	-0.1702 to 1.167
VEGF-A	P-value	0.0019	>0.9999	<0.0001	>0.9999	0.7412	>0.9999	>0.9999	>0.9999	0.0011	0.4757
		**	ns	****	ns	ns	ns	ns	ns	**	ns
	95.00 % CI	-6.407 to -1.257	-2.316 to 2.834	-11.36 to - 6.209	-2.427 to 2.723	-4.076 to 1.075	-2.352 to 2.799	-3.342 to 1.809	-2.340 to 2.811	1.492 to 6.643	-0.8395 to 4.311

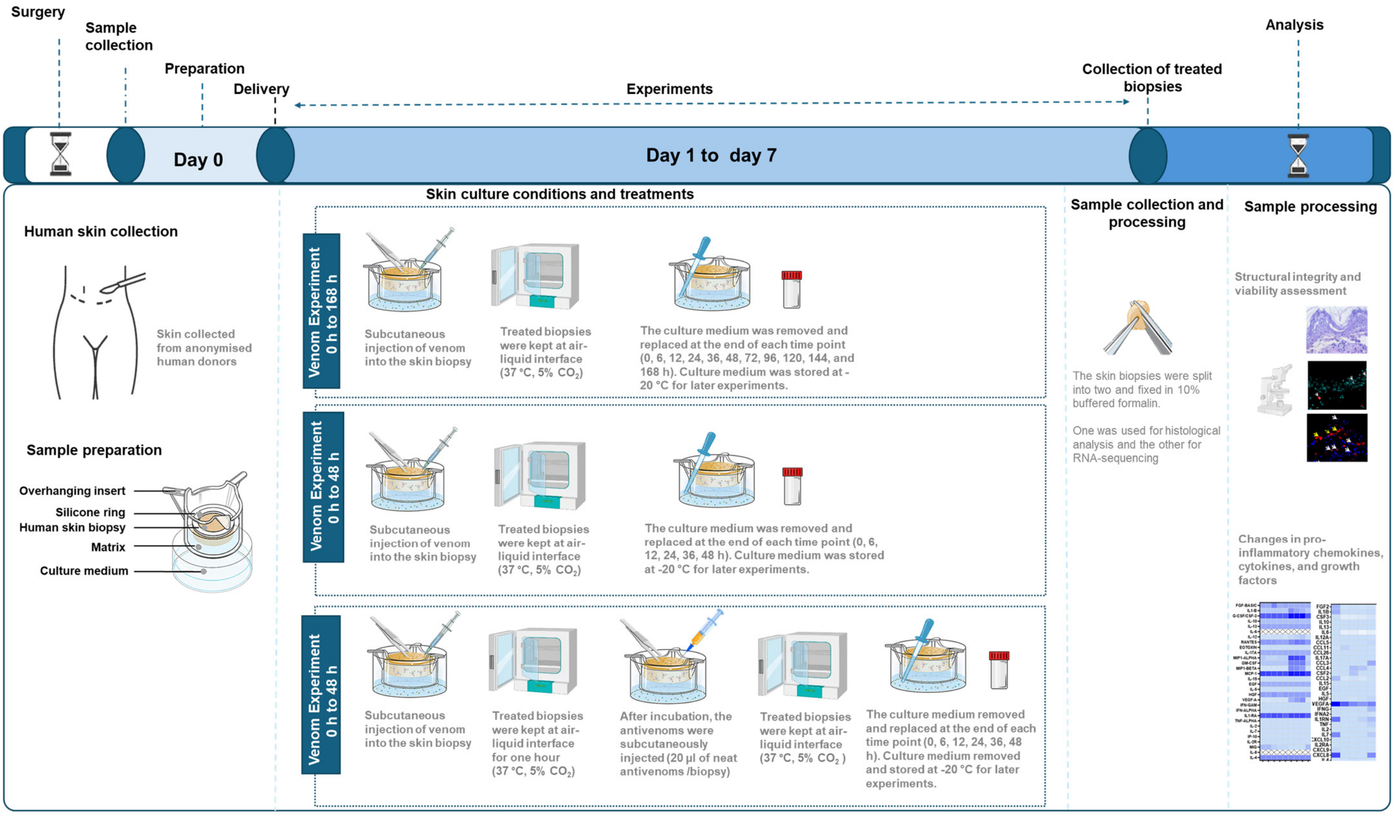

Figure S1. Schematic overview of ex vivo human skin model experiments following the subcutaneous injection of snake venoms and antivenom interventions. Human skin was collected from anonymised donors (female, aged 40 and 47) and prepared by placing in a nourishing matrix and integrated into multi-well culture plates. Hyposkin® biopsies, 15 mm in diameter and 10 mm thick, were subcutaneously injected (20 µl) with *Echis ocellatus* (120 µg), *Bitis arietans* (195 µg), *Naja nigricollis* (165 µg), or Naja *haje* (156 µg) venoms for three experimental conditions: i) venom only time course from 0 h to 168 h; ii) venom only time course from 0 h to 48 h; and iii) venom with an antivenom intervention from 0 h to 48 h. Following venom treatments, biopsies were incubated at 37°C, 5% CO_2. Culture medium was removed and replaced at each time point. After incubation, culture medium was removed and stored at -20 °C for later experimental work. For the antivenom intervention experiment, one hour after venom subcutaneous injections, 20 µl of antivenom (SAIMR Echis monovalent 52 mg/ml or SAIMR polyvalent 103 mg/ml) was subcutaneously injected. For sample collection time points, skin biopsies were split in two and one part fixed in 10% buffered formalin for 48 h. The fixed biopsy was used for

histological analysis, and the unfixed biopsy for RNA-sequencing. Structural integrity, cell viability, and expression of pro-inflammatory markers were analysed from the samples collected at each timepoint.

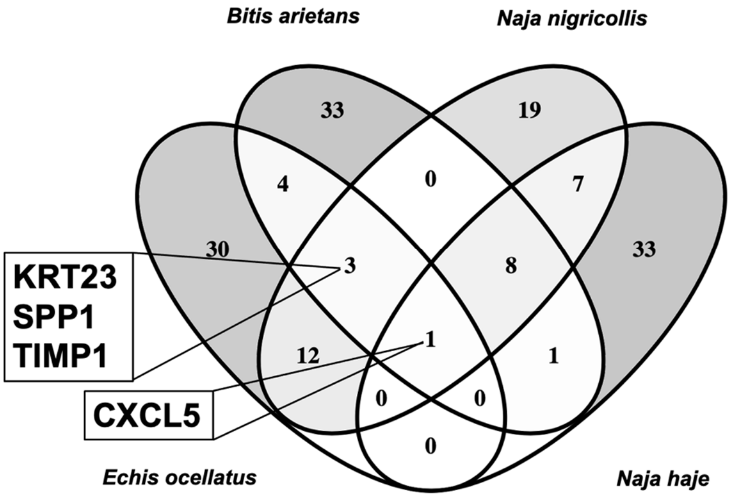

Figure S2. Upregulated gene set overlap in biopsies 48 h after subcutaneous injection of venoms. The top 50 upregulated genes from each venom (*E. ocellatus* 120 µg; *B. arietans* 195 µg; *N. nigricollis* 165 µg; N. *haje* 156 µg) injected biopsy at 48 h were used for the construction of a venn diagram in Venny 2.1 [84].

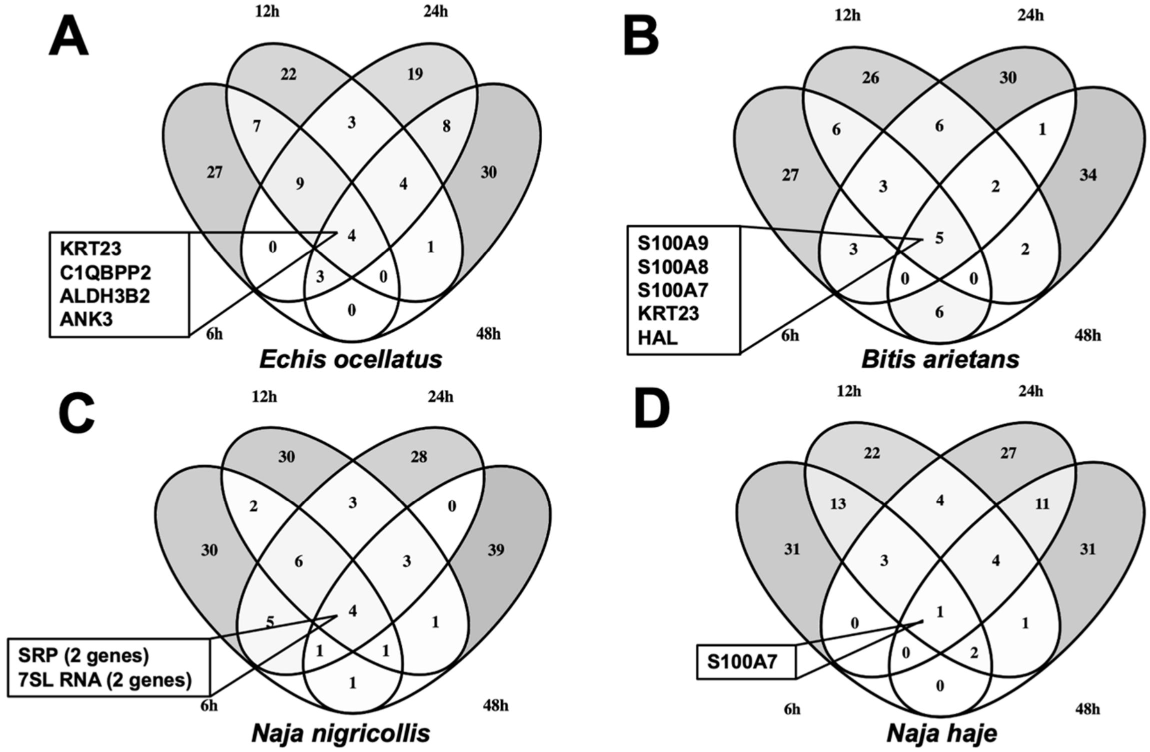

Figure S3. Upregulated gene set overlap in biopsies 6, 12, 24 and 48 h after subcutaneous injections of venoms. The top 50 upregulated gene sets for each venom, A) *Echis ocellatus* (120 µg), B) *Bitis arietans* (195 µg), C) *Naja nigricollis* (165 µg), and D) *N. haje* (156 µg) at each time point were used for constructing venn diagrams in Venny 2.1 [84].

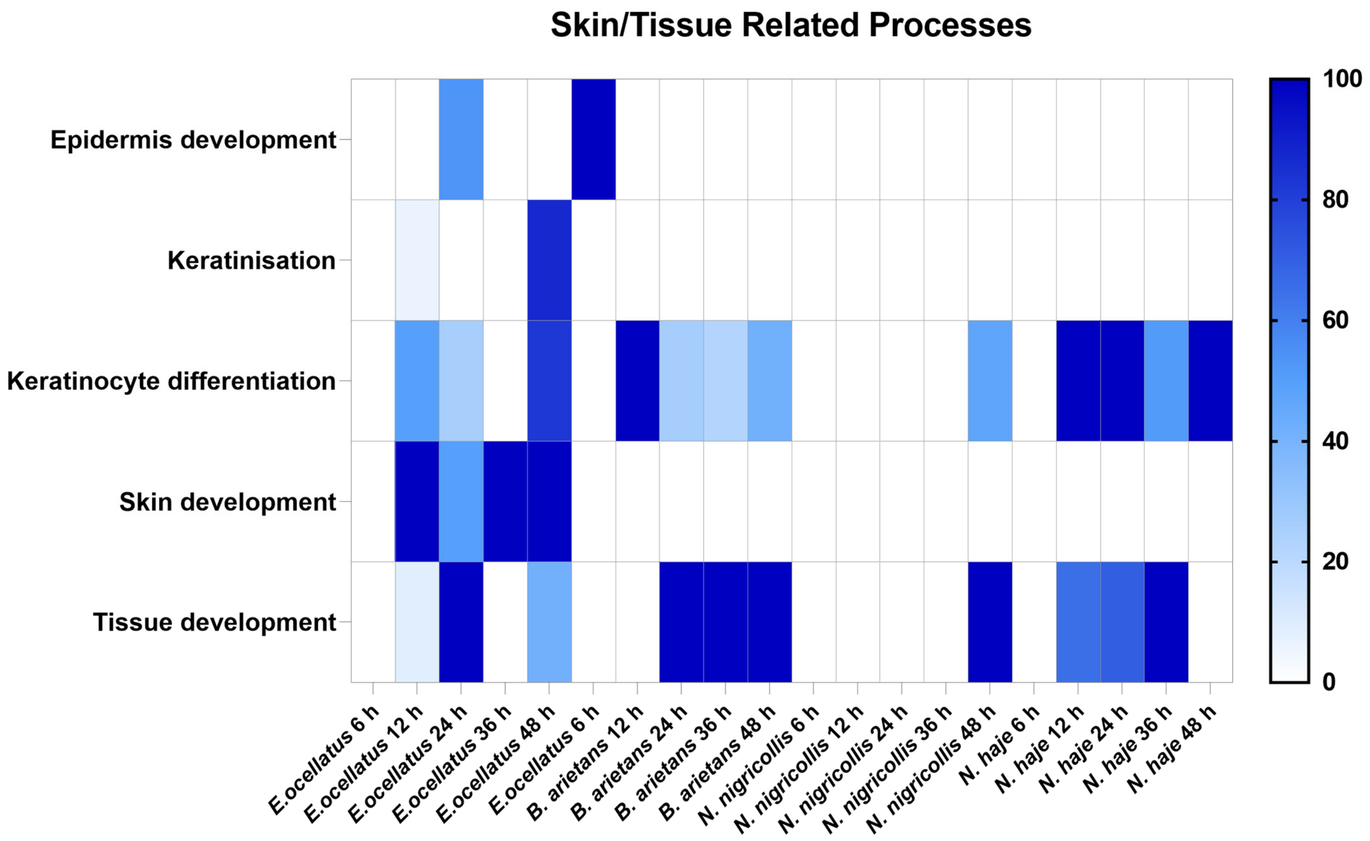

Figure S4. Enriched biological processes relating to skin identified in gene ontology analysis. Genes upregulated at least 2-fold were evaluated with the Gene Functional Annotation Tool from the DAVID Bioinformatics Database. Negative log values are reported for p-values to highlight significance.
